# Comparative efficacy of melatonin and glutathione in mitigating carboplatin-induced ovarian toxicity in rats

**DOI:** 10.1016/j.clinsp.2026.100902

**Published:** 2026-03-12

**Authors:** Halime Tozak Yıldız, Kübra Tuğçe Kalkan, Özge Cengiz Mat, Eda Köseoğlu, Özge Göktepe, Gözde Özge Önder, Arzu Yay

**Affiliations:** a Department of Histology and Embryology, Faculty of Medicine, Kirsehir Ahi Evran University, Kirsehir, Türkiye; b Department of Histology and Embryology, Faculty of Medicine, Erciyes University, Melikgazi, Kayseri, Türkiye

**Keywords:** Melatonin, Glutathione, Carboplatin, Ovarian toxicity, Antioxidant therapy, Chemotherapy-induced gonadotoxicity

## Abstract

**Objective:**

This study aimed to compare the comparative protective effects of Melatonin (Mel) and Glutathione (GSH) against Carboplatin (CARB)-induced ovarian toxicity in rats, with a focus on folliculogenesis, oxidative stress, inflammation, and apoptosis.

**Methods:**

Female Wistar rats were allocated into six groups: Control, GSH, Mel, CARB, GSH+CARB, and Mel+CARB. Mel and GSH were administered intraperitoneally for 7 days before CARB injection. Ovarian tissues were subjected to histopathological and morphometric analyses, while serum oxidative stress markers (MDA, SOD, CAT, GSH-Px), proinflammatory cytokines (TNF-α, IL-6), and AMH levels were quantified. Caspase-3 and NF-κB expressions were evaluated immunohistochemically.

**Results:**

CARB caused severe ovarian injury characterized by marked follicular depletion, stromal edema, hemorrhage, inflammation, fibrosis, and a significant reduction in preantral and Graafian follicles, with a concomitant increase in atretic follicles (p < 0.01). Both Mel and GSH pre-treatment markedly reduced histological damage, restored follicular counts, improved antioxidant status, and suppressed inflammatory and apoptotic responses. Melatonin tended to exert stronger protective effects on certain parameters, particularly in preserving AMH levels, attenuating fibrosis, and reducing Caspase-3 and NF-κB expression, although the differences between Mel+CARB and GSH+CARB groups were not statistically significant in all comparisons.

**Conclusion:**

Pre-treatment with melatonin or glutathione effectively mitigated CARB-induced ovarian toxicity by modulating oxidative stress, inflammation, and apoptosis. Melatonin appeared to provide relatively greater protection in some parameters, but without consistent statistical superiority over glutathione. These results underscore the potential of antioxidant-based strategies for fertility preservation during chemotherapy. Further studies with larger sample sizes and direct comparative analyses are warranted to clarify relative efficacy and confirm translational relevance.

## Highlight

• Both melatonin and glutathione significantly reduce carboplatin-induced ovarian damage.
• Melatonin demonstrates stronger trends in preserving follicular reserve and reducing inflammation.
• Both antioxidants restore oxidative balance and suppress apoptotic pathways.
• Antioxidant pre-treatment shows potential for fertility preservation during chemotherapy.

## Introduction

Carboplatin (CARB) is a widely used platinum-based chemotherapeutic agent with a broad spectrum of clinical activity against various malignancies, including gynecological cancers, germ cell tumors, head and neck cancers, and bladder cancer.^1^ Although structurally related to cisplatin, carboplatin has a significantly lower toxicity profile.^2^ Approved by the U.S. Food and Drug Administration (FDA), carboplatin is frequently used as part of combination chemotherapy regimens rather than as a single agent. Like cisplatin, it contains two chloride ligands, which are released within the cellular environment and interact with DNA to form intra- and interstrand cross-links.^3^ These DNA adducts trigger multiple cellular responses, including DNA damage recognition and repair, cell cycle arrest, and activation of apoptotic signaling pathways, ultimately inhibiting cell proliferation.^4^

Ovarian cancer remains one of the leading causes of mortality among gynecological malignancies, with epithelial ovarian carcinoma accounting for more than 90% of all cases.^5^ The current standard therapy involves primary cytoreductive surgery followed by platinum-based combination chemotherapy administered as soon as possible after surgery.^6^ However, a major limitation of these treatments is the dose-dependent toxicity associated with platinum compounds, which can result in significant off-target tissue injury.^7^ It is well established that Reactive Oxygen Species (ROS) generated during the metabolism of platinum-based agents contribute to this toxicity, particularly in the reproductive system. In experimental models, suppression of spermatogenesis in the testes and degeneration of primordial follicles and granulosa cells in the ovaries are considered hallmark features of platinum-induced gonadotoxicity.^8^

Chemotherapeutic agents have been shown to deplete intracellular Glutathione (GSH), one of the major endogenous antioxidants, while simultaneously increasing Myeloperoxidase (MPO) activity, an indicator of inflammatory response, and elevating Malondialdehyde (MDA), a marker of lipid peroxidation.^9^ Co-administration of antioxidants during chemotherapy has therefore been proposed as a strategy to mitigate oxidative stress-induced tissue injury and improve therapeutic tolerance. GSH is one of the body's principal antioxidant defense molecules, playing a crucial role in the detoxification of cytotoxic compounds and reactive oxygen species.^10^ Intracellular GSH concentrations differ among tissues, typically ranging from 1 to 10 mM, with the highest levels observed in the liver.^11^ Experimental supplementation of GSH in animal models has been shown to reduce hepatocyte apoptosis, lower plasma AST and ALT activities, and protect against microvascular reperfusion injury.^12^

Melatonin (Mel) is a hormone primarily secreted by the pineal gland, but it is also synthesized in various tissues, including the ovaries, retina, lens, bone marrow, and gastrointestinal tract.^13^ In the retina and gastrointestinal system, it plays roles in visual adaptation and oxidative protection, respectively. By synchronizing the body's physiology with the photoperiod, melatonin helps regulate circadian rhythms.^14^ Certain pharmacological agents can influence pineal gland activity and alter plasma melatonin concentrations. Due to its lipophilic nature, Mel easily penetrates cell membranes and acts as a potent antioxidant by scavenging hydroxyl and peroxyl radicals.^15^ This antioxidant effect is mediated through increased activity of Glutathione Peroxidase (GSH-Px) and Superoxide Dismutase (SOD), prevention of Catalase Reduction (CAT), and inhibition of Nitric Oxide Synthase (NOS). Moreover, it reduces free radical production by suppressing cytochrome P450 enzyme activity.^16^ Growing evidence indicates that melatonin exerts significant protective effects on reproductive tissues through its potent antioxidant and anti-apoptotic properties.^17^, ^18^, ^19^ Experimental studies in both female and male models have shown that melatonin reduces oxidative stress markers, preserves follicular and testicular structure, and ameliorates apoptosis-related damage.^18^

In this study, the potential protective effects of Mel and GSH were investigated against CARB-induced ovarian damage in rats. Considering the well-documented gonadotoxicity of platinum-based chemotherapeutics and the growing evidence supporting the antioxidative and anti-inflammatory properties of MEL and GSH, this study aims to explore a novel therapeutic strategy to mitigate chemotherapy-related ovarian toxicity. Enhancing the therapeutic index of CARB through such protective agents may pave the way for safer and more effective treatment options for various malignancies, particularly in reproductive-age patients where fertility preservation is a significant concern.

## Methods

### Animals and experimental groups

The study was started with twelve-week-old female Wistar albino rats. The rats were kept in a 25°C room with a 12-hour light/dark cycle, free access to water, and a standard diet from the Experimental and Clinical Research Center at *Erciyes University in Kayseri, Turkey*. The animals were assigned to six experimental groups (n = 7 per group) using stratified randomization to balance body weights across groups at baseline.

Six distinct categories of rats were set up: Control (Control), Glutatyone (GSH), Melatonin (Mel), Carboplatin (CARB) Carboplatin+Glutatyone (CARB+GSH), and Carboplatin+Melatonin (CARB+Mel). Rats in the control group were untreated. CARB (Kocak Pharma, 50 mg/45 mL. Istanbul, Turkey) was administered intraperitoneally (i.p.) at a dose of 45 mg/kg^20^ to the respective animal groups on day-7 of the experimental protocol. GSH (Sigma, Cat No; D44936, Saint Louis, USA) and Mel (M 5250; Sigma, St Louis, MO, USA) were administered to the respective groups by i.p. at doses of 20 mg/kg^21^ and 10 mg/kg^22^ for 7 days, respectively. The doses were selected from previous studies that demonstrated protective efficacy without toxicity (Mel) or included dose-response evaluation (GSH). Until the end of the experiment, no animals were lost. On the 8th day of the study, rats were sacrificed by cervical dislocation under light anesthesia. All histopathological and immunohistochemical evaluations were performed by two independent observers blinded to the group assignments. No animals were excluded during the study, and all were included in the final analyses.

The experiments were conducted in accordance with the ARRIVE guidelines (Animal Research: Reporting of In Vivo Experiments). All experimental protocols were approved by the Local Ethic Committee of Animal Experiments of Erciyes University (approval n° 22/176 date: 08.08.2022) in Turkiye.

### Ovarian morphology evaluation

The left ovary was fixed in 10% neutral formalin for 72 hours, treated with ethanol, and then embedded in paraffin blocks for histological examinations. After cutting and mounting on a slide, a serial section with a thickness of 5 μm was obtained. Following being deparaffinized with xylene, sections were rehydrated and stained with either Masson Trichrome (MT) or Hematoxylin and Eosin (H&E) to evaluate the histology. Using an Olympus BX51 microscope (Tokyo, Japan) equipped with a DP-71 camera, sections have been examined and photographed on camera.

Histopathological evaluation was performed using a semi-quantitative scoring system previously described in the literatüre.^23^ Each slide was examined independently by two blinded histologists who were unaware of the experimental groups. The following parameters were evaluated and scored on a scale from 0 to 3: 1- Follicular degeneration, 2- Inflammation, 3- Hemorrhage, 4- Edema, 5- Vascular congestion. The scoring criteria were defined as follows: 0 = None, 1 = Mild, 2 = Moderate, 3 = Severe. For each animal, ten randomly selected fields were assessed, and the mean score was calculated to represent the group value.

### Follicle morphological classification and counting

Ovarian follicles were counted on serial sections of ovarian tissue from each experimental group. To ensure representative sampling, the ovary was systematically sectioned. After the initial appearance of ovarian tissue, ten consecutive sections were collected, and the 1st, 5th, and 10th of these were selected for hematoxylin-eosin staining and histological evaluation. The remaining sections were discarded. This cycle of collecting and sampling ten sections was repeated throughout the entire ovary. Follicles were classified by two independent histologists into primordial, primary, preantral, antral, and atretic stages based on established morphological criteria, primarily the number and shape of granulosa cell layers. Healthy follicles were identified by an intact basal lamina, a visible oocyte with a germinal vesicle, and a distinct nucleolus, while atretic follicles were characterized by pyknotic nuclei and degenerating granulosa cells.^23^

### Immunohistochemistry

The immunohistochemical staining kit (Thermo Ultravision Detection System, Fremont, USA, TP-125-HL) was used in conjunction with the streptavidin-biotin-peroxidase method. This method was used to identify the expressions of Caspase-3 (Cas-3), Nüclear Faktör-κB (NF-κB), and Anti Mullerian Hormone (AMH) in ovarian tissues. Five micron-thick cross-sections of ovarian tissue blocks were cut, deparaffinized, rehydrated, and then washed at room temperature in Phosphate-Buffered Saline (PBS). To prevent endogenous peroxidase activity, 3% hydrogen peroxide was applied for 5-minutes. They were rewashed with PBS and then allowed to recover antigens in 10% sodium citrate buffer (pH 6.0) for 5 minutes at 95°C in the microwave oven. They were subsequently allowed to rest at room temperature for a total of 20 minutes. Primary antibodies cleaved Cas-3 (Cell Signaling Technology, Cat. n° 7938, 1:500), NF-κB (Cell Signaling Technology, Cat.n° 8242, 1:800), and AMH (Santa Cruz Bıotechnology, Oregon, ABD-sc 1667529; 1:150) were treated with the sections for an entire night at 4°C. The sections were treated with biotinylated secondary antibodies. After washing, apply the streptavidin peroxidase complex and utilize DAB (Diaminobenzidin echromogen and substrate system, Thermo Fisher Scientific, Waltham, Massachusetts, USA). Mayer's hematoxylin was applied to counterstain it. For each part of the ovaries, images were captured from 10 separate locations using a digital camera (DP71) set on an Olympus BX51 light microscope (Tokyo, Japan). Using the Image J software program (NIH; Washington, U.S.A.), immunoreactivity densities of Cas-3, NF-κB and AMH were determined.

### Enzyme‑linked immunosorbent assay

The right ovary was promptly bisected on an ice-cold mold. Ovarian samples were transferred into Eppendorf tubes and stored at -80°C for preservation. For biochemical analyses, ovarian tissues were transferred into phosphate-buffered saline (PBS; 0.01 M, pH 7.4) for homogenate preparation. The homogenized samples were then centrifuged at 4°C for 10 minutes at 1500 × g. The resulting supernatants were aliquoted and used for Enzyme-Linked Immunosorbent Assay (ELISA) analyses.

To assess the antioxidant and AMH capacity of the ovarian tissue, the levels of GSH-Px (Sunred Bio, Cat. n° 201-11-5104; Shanghai, China), SOD, and CAT (Sunred Bio, Cat. n° 201-11-5106; Shanghai, China) were measured using commercial ELISA kits. Additionally, lipid peroxidation levels were evaluated by quantifying MDA concentrations (Sunred Bio, Cat. n° 201-11-0157; Shanghai, China).

### Statistical analyses

Statistical analyses were performed using GraphPad Prism version 9.0 (GraphPad Software, San Diego, CA, USA). The Shapiro-Wilk test was used to evaluate the normality of data distribution. For comparisons involving more than two groups, one-way analysis of variance (ANOVA) was applied to normally distributed data, followed by Bonferroni’s post-hoc correction to control for multiple comparisons. For non-parametric data, the Kruskal-Wallis test was used, followed by Dunn’s multiple comparison test. These post-hoc adjustments were performed to minimize the risk of Type I error due to multiple testing. All data are presented as mean ± Standard Deviation (SD) or median (interquartile range), as appropriate. A p-value < 0.05 was considered statistically significant.

### Post-hoc power analysis

A post-hoc power analysis was conducted based on the NF-κB immunohistochemical data, which represented one of the main outcome measures. Using a one-way ANOVA model (α = 0.05, six groups, n = 7 per group equivalent), the observed effect size (Cohen’s *f*) was 1.59, corresponding to an η² value of 0.72. According to this analysis, the achieved statistical power was 0.98, indicating that the current sample size was sufficient to detect significant group differences. The power analysis was performed using GPower version 3.1 (Heinrich-Heine University, Düsseldorf, Germany).

## Results

### Ovarian histoarchitecture is preserved by melatonin and glutatione against carboplatin-induced damage

**Fig. 1:**
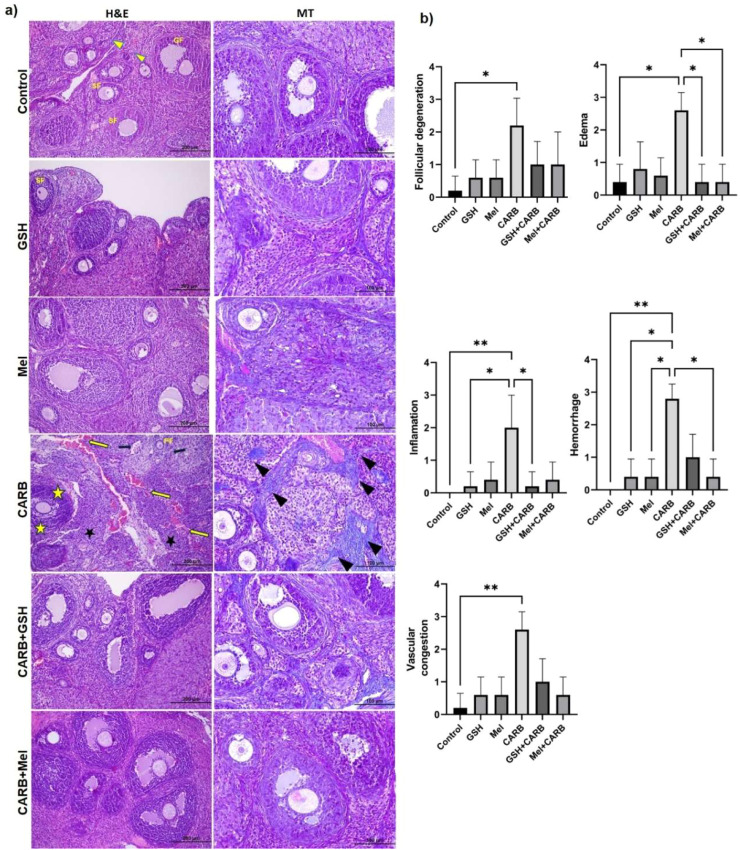
(a) Representative photomicrographs of Hematoxylin & Eosin and Masson’s trichrome staining (Olympus BX51, Tokyo, Japan. H&E; × 100: Scale bar; 200 µm, MT; × 200: Scale bar; 100 µm). In the Control group, normal ovarian morphology was observed, with well-preserved primordial (yellow arrowhead), primary, secondary, and Graafian follicles. No signs of degeneration, inflammation, or fibrosis were detected. In the GSH and Melatonin (Mel) groups, follicular structures were preserved, and stromal areas appeared normal. No degeneration or inflammation was observed.In the CARB group, follicular atresia, cytoplasmic vacuolization, nuclear pyknosis, and degenerative changes in granulosa cells were evident (yellow star). Vascular congestion (yellow arrow), dense cellular infiltration (black star), hemorrhage (black arrow), and intense fibrosis (black arrowhead) were observed in the stromal tissue. In the GSH+CARB and Mel+CARB groups, mild follicular degeneration and stromal congestion were noted. Partial preservation of follicular structures was observed. (b) Comparison of ovarian histoscoring results. Statistically significant differences between groups are indicated by asterisks in each graph: * p < 0.05, ** p < 0.01, * p < 0.001.

To evaluate the protective effects of Mel and GSH against CARB-induced ovarian damage, these two antioxidant agents were administered for one week prior to CARB treatment. Histopathological analysis revealed that the CARB-only group exhibited marked follicular degeneration (p < 0.05), stromal edema (p < 0.05), vascular congestion (p < 0.01), hemorrhage (p < 0.01), inflammation (p < 0.01), and fibrosis (p < 0.05). Semi-quantitative histological scoring showed that the CARB group had significantly higher scores across all parameters compared to the control group. In contrast, pre-treatment with antioxidants in the Mel+CARB and GSH+CARB groups markedly reduced the severity of tissue damage. In the Mel+CARB group, the degrees of edema, hemorrhage, and fibrosis were significantly lower than those in the CARB group (p < 0.05). Similarly, in the GSH+CARB group, significant reductions in edema and inflammation were observed (p < 0.05). In the groups treated with Mel or GSH alone, the morphological integrity of the ovarian tissue was preserved, similar to the control group, and no evidence of inflammatory response, vascular disruption, or fibrotic changes was detected (Fig. 1a‒b, Table 1).

**Table 1:** Histopathological scoring of ovarian tissue in experimental groups.

Histoscoring	Control	GSH	Mel	CARB	GSH+CARB	MEL+CARB	p
Follicular degeneration	0.0 (0.0–1.0)	0.5 (0.0–1.0)	0.5 (0.0–1.0)	2.0 (1.0–3.0)	1.0 (0.0–2.0)	1.0 (0.0–2.0)	0.013^a^
Edema	0.5 (0.0–1.0)	0.5 (0.0–2.0)	0.5 (0.0–1.0)	2.5 (2.0–3.0)	0.5 (0.0–1.0)	0.5 (0.0–1.0)	0.031^a,b,c^
Inflammation	0.0 (0.0–1.0)	0.2 (0.0–1.0)	0.5 (0.0–1.0)	2.0 (1.0–3.0)	0.2 (0.0–1.0)	0.4 (0.0–1.0)	0.090^a,b^
Hemorrhage	0.0 (0.0–0.0)	0.4 (0.0–1.0)	0.4 (0.0–1.0)	2.8 (2.0–3.0)	1.0 (1.0–2.0)	0.4 (0.0–1.0)	0.002^a,c^
Vascular congestion	0.2 (0.0–1.0)	0.6 (0.0–1.0)	0.6 (0.0–1.0)	2.6 (2.0–3.0)	1.0 (1.0–2.0)	0.6 (0.0–1.0)	0.006^a^
Fibrosis	0.2 (0.0–1.0)	0.4 (0.0–1.0)	0.4 (0.0–1.0)	2.6 (2.0–3.0)	0.4 (0.0–1.0)	0.2 (0.0–1.0)	0.008^a,c^

Data are expressed as median (min–max) values, as the variables did not follow a normal distribution according to the Shapiro-Wilk test. Statistical differences among groups were analyzed using the Kruskal-Wallis test, followed by Dunn’s multiple comparison test for post-hoc analysis. Different superscript letters (a, b, c) indicate statistically significant differences between groups (p < 0.05).

### Melatonin and glutathione mitigate follicular depletion caused by carboplatin

When the stages of follicular development in ovarian tissue were compared between groups, statistically significant differences were observed in the numbers of primary, preantral, graafian, and atretic follicles. The number of primary follicles was significantly decreased in the CARB group (p < 0.01). However, in the Mel+CARB and GSH+CARB groups, the number of primary follicles was significantly higher than in the CARB group (p < 0.05).

The number of preantral follicles was also significantly reduced in the CARB group (p < 0.01). In the Mel+CARB group, a significant increase was observed compared to the CARB group (p < 0.05), whereas the GSH+CARB group showed a similar but non-significant trend toward improvement.

The number of graafian follicles was markedly reduced in the CARB group (p < 0.01). Pre-treatment with melatonin or glutathione significantly prevented this reduction, as both the GSH+CARB and Mel+CARB groups exhibited significantly higher counts compared with the CARB group (p < 0.01).

**Fig. 2:**
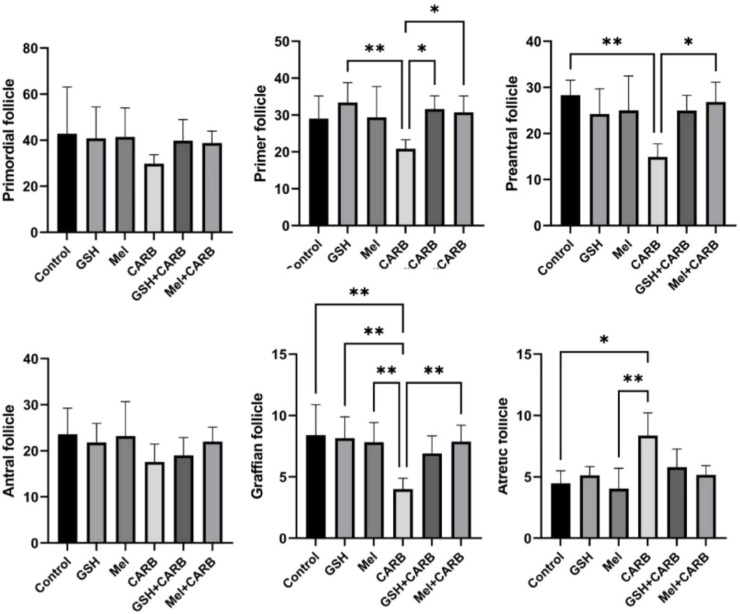
Quantitative evaluation of ovarian follicle counts at different developmental stages in experimental groups. The bar graphs represent the mean ± SD number of primordial, primary, preantral, antral, graafian, and atretic follicles in ovarian tissues from control, GSH, Mel, CARB, GSH+CARB, and Mel+CARB groups. * p < 0.05, ** p < 0.01.

Conversely, the number of atretic follicles was significantly increased in the CARB group compared to the control (p < 0.01). However, in both the GSH+CARB and Mel+CARB groups, atretic follicle counts were significantly reduced and approached control levels (p < 0.05 and p < 0.01, respectively). No statistically significant differences were detected among the groups with respect to primordial and antral follicle counts (Fig. 2).

### Melatonin and glutathione pre-treatment suppresses apoptotic and pro-inflammatory cytokine expression in ovarian tissue

IHC staining and quantitative analysis revealed a significant increase in apoptotic and inflammatory markers in the CARB group. In particular, Cas-3 and NF-κB immunoreactivity were significantly elevated in the CARB group compared to the control (p < 0.001). Additionally, inflammation-related proteins measured in tissue homogenates TNF-α (p < 0.01) and IL-6 (p < 0.001), were also markedly increased. These findings indicate that carboplatin induces both apoptotic cell death and a proinflammatory response in ovarian tissue.

**Fig. 3:**
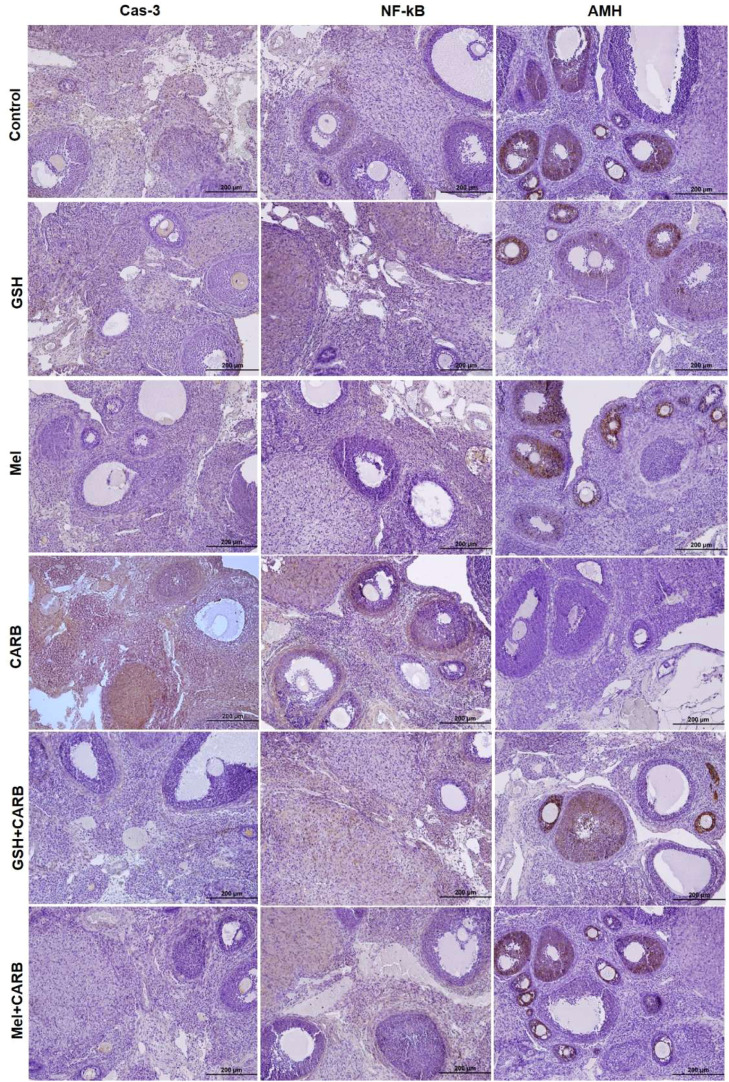
Representative photomicrographs of immunohistochemical staining for Cas-3, NF-κB, and AMH in ovarian sections from control, GSH, Mel, CARB, GSH+CARB, and Mel+CARB groups. (Olympus BX51, Tokyo, Japan. Scale bar; 200 µm). Strong Cas-3 and NF-κB staining in the CARB group indicated increased apoptosis and inflammation, while AMH expression was markedly reduced, reflecting diminished follicular reserve. Pre-treatment with melatonin or glutathione attenuated apoptotic and inflammatory responses and restored AMH immunoreactivity to near-control levels.

**Fig. 4:**
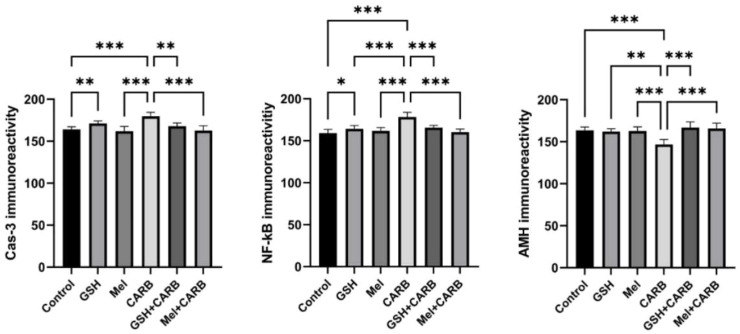
Evaluation of immünreactivity scores in ovarian tissue. Bar graphs showing the mean ± SD values for Cas-3, NF-κB, and AMH immunoreactivity scores. Statistical significance: *p < 0.05, **p < 0.01, ***p < 0.001.

**Fig. 5:**
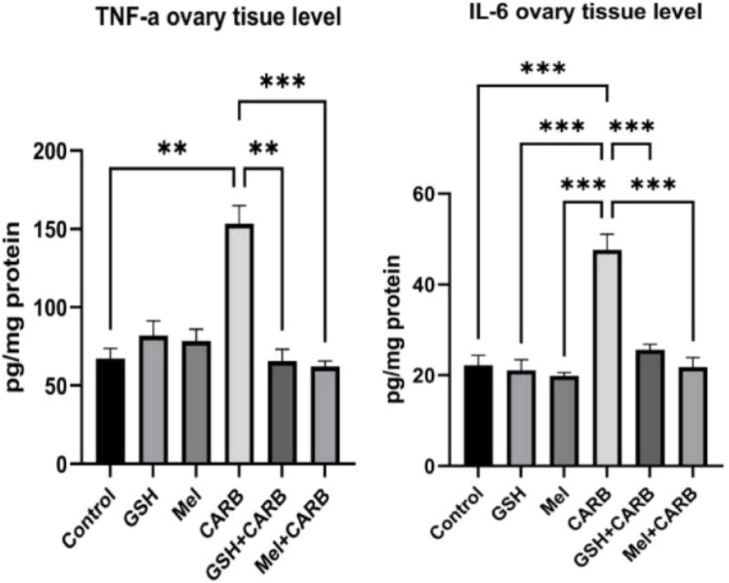
Evaluation of inflammation markers in ovarian tissue. Bar graphs showing the mean ± SD values for TNF-α, and IL-6 ovarian tisue level. Statistical significance: * p < 0.05, * p < 0.01, *** p < 0.001.

However, in both the Mel+CARB and GSH+CARB groups, the expression levels of apoptotic and inflammatory markers were significantly reduced, approaching those of the control group. Similarly, tissue levels of inflammatory cytokines were significantly decreased. This decline suggests that pre-treatment with both antioxidant agents effectively suppressed carboplatin-induced apoptosis and inflammation. Notably, in the Mel+CARB group, both Caspase-3 and NF-κB levels were significantly reduced (p < 0.01 and p < 0.001, respectively). Inflammatory cytokines were also significantly lower in the Mel+CARB group (TNF-α and IL-6: p < 0.001) and the GSH+CARB group (TNF-α: p < 0.01; IL-6: p < 0.001) compared to the CARB group (Fig. 3, 4, 5).

### Melatonin and glutathione prevented the carboplatin-induced decrease in AMH expression

The immunoreactivity of AMH was significantly reduced in the CARB group compared with the control group (p < 0.001), indicating a decline in ovarian reserve. However, in the Mel+CARB and GSH+CARB groups, AMH levels were markedly increased, and this reduction was both statistically and biologically reversed (p < 0.001). These findings suggest that melatonin and glutathione exert a protective effect on follicular reserve. (Fig. 3, 4)

### Melatonin and glutathione restore oxidative balance disrupted by carboplatin exposure

**Fig. 6:**
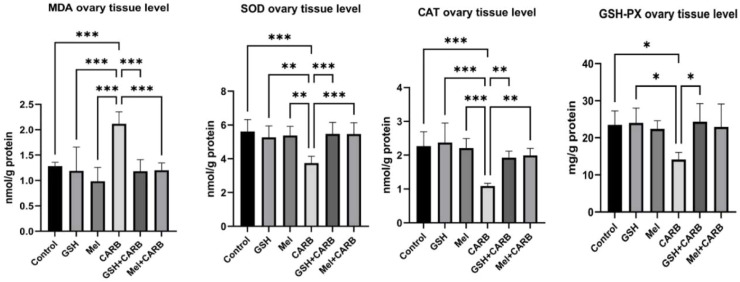
Evaluation of oxidative stress markers in ovarian tissue. Bar graphs showing the mean ± SD values for MDA, SOD, CAT, and GSH-Px ovarian tisue level. Statistical significance: * p < 0.05, * p < 0.01, *** p < 0.001.

CARB administration in ovarian tissue induced significant alterations in oxidative stress markers. MDA levels were significantly increased in the CARB group (p < 0.001), indicating enhanced lipid peroxidation and cellular oxidative damage. Conversely, the levels of antioxidant defense marker enzymes SOD, CAT, and GSH-Px were significantly reduced in the CARB group (SOD and CAT;p < 0.001, GSH-Px;p < 0.05). Pre-treatments with Mel+CARB and GSH+CARB effectively restored this imbalance. In both groups, MDA levels were significantly reduced (p < 0.001), accompanied by a notable elevation in antioxidant enzyme levels. Specifically, in the Mel+CARB group, SOD (p < 0.001) and CAT (p < 0.01) levels were significantly higher than in the CARB group, whereas in the GSH+CARB group, SOD (p < 0.001), CAT (p < 0.01), and GSH-Px (p < 0.05) were all significantly elevated (Fig. 6).

## Discussion

In the present study, the authors investigated the protective effects of melatonin and glutathione against carboplatin-induced ovarian toxicity by evaluating histopathological alterations, follicular development, apoptotic and inflammatory markers, as well as oxidative stress parameters. CARB, a widely used chemotherapeutic agent, significantly impaired ovarian histoarchitecture, reduced follicle numbers at various developmental stages, and increased atretic follicles. These alterations were accompanied by increased expression of Cas-3 and NF-κB, elevated levels of pro-inflammatory cytokines (TNF-α, IL-6), and significant oxidative stress, as evidenced by increased MDA and decreased antioxidant enzyme levels (SOD, CAT, GSH-Px). Pre-treatment with Mel and GSH notably ameliorated these adverse effects. Both agents preserved follicular structure, reduced apoptosis and inflammation, and restored antioxidant defense systems in the ovarian tissue. Furthermore, the marked decrease in AMH levels observed in the CARB group was significantly reversed by melatonin and glutathione, indicating preservation of the ovarian reserve. These findings collectively suggest that melatonin and glutathione exert protective actions through their antioxidant, anti-inflammatory, and anti-apoptotic capacities; however, the precise molecular mechanisms underlying these effects remain to be clarified in future studies.

Ovarian cancer is the sixth most common malignancy among women in developed countries and the fifth leading cause of cancer-related death, representing a complex disease associated with poor survival rates.^24^ The combination chemotherapy of Paclitaxel (PTX) and Carboplatin (CARB) is considered the first-line treatment regimen for ovarian cancer.^25^ Both agents play distinct roles in inhibiting the growth of ovarian cancer through different mechanisms,^26^ and they are known to exert a synergistic anti-tumor effect by activating separate apoptotic signaling pathways, thereby potentially reducing drug resistance.^25^

The gonadotoxic effects of platinum-based chemotherapeutic agents have been demonstrated in numerous studies, both histopathologically and in terms of follicular development.^27^, ^28^, ^29^ In the present study, CARB administration led to significant histopathological damage in ovarian tissue, including follicular degeneration, prominent stromal edema, congestion, hemorrhage, inflammation, and fibrosis, particularly in the medullary region. In addition, morphometric analysis revealed a reduction in preantral and Graafian follicles, along with an increase in atretic follicles, indicating disruption of ovarian histoarchitecture. Mel appeared to be slightly more effective than GSH in alleviating CARB-induced stromal injury, particularly in reducing edema and fibrotic changes. Furthermore, melatonin treatment tended to provide greater protection in preventing the loss of preantral and Graafian follicles, which may indicate a relatively stronger role in preserving folliculogenesis. These findings are consistent with previous studies suggesting that Mel and GSH may prevent structural damage in ovarian histoarchitecture by suppressing oxidative stress.^10^^,^^11^^,^^15^, ^16^, ^17^, ^18^^,^^21^^,^^22^ Both Mel and GSH are key endogenous antioxidant molecules that neutralize free radicals and preserve tissue integrity by limiting oxidative stress-induced cellular damage.^30^^,^^31^

It has been previously reported that platinum-based chemotherapeutic agents such as carboplatin increase intracellular ROS production, thereby triggering lipid peroxidation (as indicated by elevated MDA levels), inflammatory responses (increased TNF-α and IL-6), cellular damage, and apoptosis.^2^^,^^7^^,^^8^ In the current study, the increased immunoreactivity of Caspase-3 and NF-κB, along with significantly elevated levels of TNF-α and IL-6 in the CARB group, indicate that CARB may have simultaneously activated both apoptotic and inflammatory pathways. Although both antioxidants used as pre-treatment (Mel and GSH) mitigated the harmful effects of CARB, Mel appeared to exert comparatively stronger inhibitory effects on apoptotic (Cas-3) and inflammatory (NF-κB, TNF-α, IL-6) responses. While these observations are consistent with literature findings, they should be interpreted as indicative rather than conclusive evidence of mechanistic involvement. Mel has been shown to regulate multiple molecular pathways, including inflammation, oxidative stress, apoptosis, and autophagy, under various pathophysiological conditions.^32^

Cas-3 is the most critical executioner in the caspase family and represents the final common substrate of both intrinsic and extrinsic apoptotic pathways.^32^ Once activated, Cas-3 cleaves this substrate, thereby reflecting the progression of apoptosis.^33^ In the present study, CARB treatment appeared to initiate this process in ovarian tissue, as evidenced by intense Cas-3 immunoreactivity. In contrast, Mel and GSH may have contributed to the prevention of apoptosis, possibly through inhibition of Cas-3, Cas-9, and Bax signaling pathways. However, because these observations rely solely on immunohistochemistry, they indicate potential involvement rather than providing definitive mechanistic evidence, and molecular validation is required to confirm these pathways.

NF-κB is a key regulator of apoptosis, immunity, aging, and inflammation due to its role in the expression of proinflammatory genes, including cytokines, chemokines, and adhesion molecules.^34^ In the present study, the absence of elevated NF-κB, TNF-α, and IL-6 levels in melatonin- and glutathione-treated groups suggests potential modulation of inflammatory responses by these agents. However, these findings represent associative correlations rather than direct mechanistic proof. During the early stages of inflammation, melatonin functions transiently as a proinflammatory agent for 2–3 hours, which is necessary for adaptation to acute stress.^35^ However, in later stages, it suppresses inflammation by inhibiting the binding of NF-κB to DNA and downregulating Cyclooxygenase-2 (COX-2) expression.^36^ Similarly, intracellular GSH levels typically decrease during early inflammation, which can trigger NF-κB activation and promote the production of essential proinflammatory cytokines. Nevertheless, pharmacological maintenance or restoration of GSH levels can inhibit NF-κB signaling and cytokine release, thereby preventing excessive or chronic inflammation.^37^ Thus, although the present IHC findings suggest possible modulation of NF-κB–related pathways, definitive mechanistic interpretation requires molecular-level confirmation through gene or protein expression analyses.

AMH is a well-established biomarker of ovarian reserve and is commonly used to assess the gonadotoxic effects of chemotherapy on the ovaries.^23^^,^^27^^,^^38^ In the present study, CARB administration significantly reduced AMH expression; however, both MEL and GSH treatments effectively restored AMH levels. Notably, melatonin treatment resulted in a more prominent increase in AMH expression compared to GSH, which may indicate a relatively stronger effect on folliculogenesis. This finding indicates that MEL may exert more prominent effects on folliculogenesis. Nonetheless, both agents successfully counteracted the CARB-induced reduction in AMH, thereby contributing to the maintenance of follicular reserve.

Mel is a pineal hormone with lipophilic properties, allowing it to easily cross cell membranes and exert antioxidant effects through both direct and indirect mechanisms.^39^ Previous studies have reported that melatonin not only scavenges ROS directly but also enhances the expression of antioxidant enzymes such as SOD, CAT, and GSH-Px, thereby providing indirect antioxidant defense.^17^^,^^40^ Melatonin is an indoleamine with notable antioxidant properties, making it a promising agent to counteract inflammatory and neoplastic processes and to manage transplantation outcomes.^18^^,^^41^ However, despite strong evidence from animal studies supporting its antioxidant mechanisms, the clinical applications of melatonin in reproductive medicine are still quite limited in the literature.^18^^,^^19^ Similarly, GSH is an endogenous antioxidant capable of neutralizing free radicals and supporting the GSH-dependent peroxidase system. GSH is a tripeptide (γ-L-glutamyl-L-cysteinyl-glycine) and is considered the predominant non-protein thiol in mammalian cells, working alone or in concert with enzymes to reduce superoxide radicals, hydroxyl radicals, and peroxynitrites.^42^ The intracellular balance and optimal concentration of GSH, as well as its redox ratio, are critical components of redox homeostasis in healthy cells.^43^ In this study, both melatonin and GSH administration were found to restore the disrupted levels of GSH-Px, SOD, and CAT in the CARB-treated group, supporting their role in maintaining antioxidant defense; however, the specific upstream molecular regulators of this effect warrant further investigation.

## Conclusion

In this study, the protective effects of melatonin and glutathione against carboplatin-induced ovarian damage were comprehensively evaluated at histopathological, morphometric, biochemical, and immunohistochemical levels. The findings show that both melatonin and glutathione significantly attenuate ovarian injury by restoring oxidative balance and suppressing apoptotic signaling pathways. Consistent with the observed trends, melatonin demonstrated comparatively stronger efficacy, particularly in preserving the follicular reserve, enhancing AMH expression, and reducing inflammatory markers. Overall, the ability of both antioxidants to mitigate carboplatin-induced ovarian toxicity highlights their potential as pre-treatment strategies for fertility preservation during chemotherapy. A major limitation of the present study is its single-dose carboplatin model, which does not adequately represent the repeated-cycle exposure used in clinical oncology. To enhance clinical extrapolation, future work should include multi-cycle regimens, varied dosing schedules, and functional fertility outcomes such as estrous cyclicity and mating performance.

## Clinical trial number

Not applicable.

## Ethics approval and Institutional Review Board statement

The rats were obtained and accommodated at the Experimental and Clinical Research Center of Erciyes University. The study was designed and conducted in compliance with the ARRIVE guidelines for in vivo animal research. Erciyes University Animal Experiments Local Ethics Committee approved the experimental guidelines (decision n° 22/176 date: 08.08.2022).

## Data availability statement

The datasets generated and/or analyzed during the current study are available from the corresponding author upon reasonable request.

During the preparation of this work, the author(s) used the free-access chat version of OpenAI's GPT-5 model in order to assist with the translation of the manuscript. After using this tool/service, the author(s) reviewed and edited the content as needed and take(s) full responsibility for the content of the publication.

## Authors' contributions

Conception and design: HTY, AY, GÖÖ. Collection and assembly of data: KTK, ÖG, ÖCM, EK. Data analysis and interpretation: HTY, KTK, ÖCM, EK. Manuscript writing: HTY. Editing and final approval of manuscript: All authors. Use of AI tools: Artificial Intelligence (AI) assistance (GPT-5, OpenAI) was used only for improving the English language, grammar, and phrasing of the manuscript. All scientific content, interpretations, and conclusions were entirely written, reviewed, and verified by the authors.

## Funding

Kırşehir Ahi Evran University Scientific Research Project. Project n° TIP.A2.25.014.

## Conflicts of interest

The authors declare no conflicts of interest.
